# Testing the Generalist-Specialist Dilemma: The Role of Pyrrolizidine Alkaloids in Resistance to Invertebrate Herbivores in *Jacobaea* Species

**DOI:** 10.1007/s10886-015-0551-4

**Published:** 2015-02-11

**Authors:** Xianqin Wei, Klaas Vrieling, Patrick P. J. Mulder, Peter G. L. Klinkhamer

**Affiliations:** 1 Plant Ecology and Phytochemistry, Institute of Biology, Leiden University, Sylviusweg 72, P.O. Box 9505, 2300 RA Leiden, The Netherlands; 2 RIKILT-Wageningen UR, Wageningen University and Research Center, P.O. Box 230, 6700 AE Wageningen, The Netherlands

**Keywords:** Secondary metabolites diversity, F2 hybrids, *Deroceras invadens*, *Longitarsus jacobaeae*, *Jacobaea vulgaris*, Feeding damage

## Abstract

Plants produce a diversity of secondary metabolites (SMs) to protect them from generalist herbivores. On the other hand, specialist herbivores use SMs for host plant recognition, feeding and oviposition cues, and even sequester SMs for their own defense. Therefore, plants are assumed to face an evolutionary dilemma stemming from the contrasting effects of generalist and specialist herbivores on SMs. To test this hypothesis, bioassays were performed with F2 hybrids from *Jacobaea* species segregating for their pyrrolizidine alkaloids (PAs), using a specialist flea beetle (*Longitarsus jacobaeae*) and a generalist slug (*Deroceras invadens*). Our study demonstrated that while slug feeding damage was negatively correlated with the concentration of total PAs and that of senecionine-like PAs, flea beetle feeding damage was not affected by PAs. It was positively correlated though, with leaf fresh weight. The generalist slug was deterred by senecionine-like PAs but the specialist flea beetle was adapted to PAs in its host plant. Testing other herbivores in the same plant system, it was observed that the egg number of the specialist cinnabar moth was positively correlated with jacobine-like PAs, while the silver damage of generalist thrips was negatively correlated with senecionine- and jacobine-like PAs, and the pupae number of generalist leaf miner was negatively correlated with otosenine-like PAs. Therefore, while the specialist herbivores showed no correlation whatsoever with PA concentration, the generalist herbivores all showed a negative correlation with at least one type of PA. We concluded that the generalist herbivores were deterred by different structural groups of PAs while the specialist herbivores were attracted or adapted to PAs in its host plants.

**Electronic supplementary material:**

The online version of this article (doi:10.1007/s10886-015-0551-4) contains supplementary material, which is available to authorized users.

## Introduction

Plants have evolved a variety of defense systems that ward off or reduce attack from other organisms such as herbivores and pathogens. These defenses include secondary metabolites (SMs) as well as a great diversity of external and internal physical barriers, including trichomes, thorns and spines, lignified cell walls, and silica crystals (Bennett and Wallsgrove 1994; Lucas et al. 2000; Paré and Tumlinson 1999). As herbivores can cause severe damage resulting in a loss of fitness (Becker 1983; Blundell and Peart 2000), it is expected that plants with high levels of defensive traits are selected in nature (Geber and Griffen 2003). Among these, numerous studies covering a wide range of species have revealed that SMs constitute an important class of defense (Hay and Fenical 1988; Textor and Gershenzon 2009).

Secondary metabolites are regarded as dispensable for basic growth and development yet indispensable for the fitness and survival of an organism (Janz and Nylin 1998). The most characteristic feature of SMs is their great structural multiplicity among and within plant species (Hartmann 1996). More than 500 000 SMs have been discovered in the plant kingdom (Hadacek 2002), and their diversity even within one plant species is enormous. For instance, more than 170 SMs belonging to seven major groups have been identified in *Arabidopsis thaliana* (D’Auria and Gershenzon 2005).

Insect herbivores are considered an important driving force for the diversification of SMs due to their close long-term association with plants (Janz and Nylin 1998). There are several hypotheses on the way SM diversity can increase herbivore deterrence. One of them states that the presence of multiple herbivores selects for increased SM diversity assuming that each specific SM confers resistance against a specific herbivore or a group of specific herbivores (Lason et al. 2011). Hence, a mixture of SMs is selected for by multiple herbivores (Juenger and Bergelson 1998, 2000; Macel et al. 2005; Mithen et al. 1995).

In addition to structural diversity, SMs frequently exhibit a high genotypic variation in their concentration, probably explained by a balance between the benefits of an increased resistance against herbivores and the costs of production and storage (Coley et al. 1985; Herms and Mattson 1992). The production of SMs, however, can come with an ecological cost, since some herbivores may be attracted to plants with a high SM content (van der Meijden 1996). In fact, while generalist herbivores usually are deterred by high concentrations of SMs, specialist herbivores often are adapted to them (van Dam et al. 1995; van der Meijden 1996). Moreover, several specialist herbivores sequester SMs and use them for host plant recognition, feeding and oviposition cues, and even as nuptial gifts (Dussourd et al. 1991; Nishida 2002; Opitz and Müller 2009). Therefore, plants presumably face a dilemma due to potentially contrasting natural selection by generalist and specialist herbivores that is correlated to the production of defense-related chemicals (van der Meijden 1996). However, so far there is only limited experimental evidence for this hypothesis (Lankau 2007; Lankau and Strauss 2008).

To evaluate the role of SMs in the resistance against generalist and specialist herbivores, a series of F2 hybrid plants were fed to a major specialist herbivore flea beetle, *Longitarsus jacobaeae* (Chrysomelidae) and a common generalist slug, *Deroceras invadens* (Agriolimacidae). The hybrid plants were derived from a cross between *Jacobaea vulgaris* (common ragwort, syn. *Senecio jacobaea*) and *Jacobaea aquatica* (marsh ragwort, syn. *Senecio aquaticus*). Both species contain pyrrolizidine alkaloids (PAs), a group of typical SMs with a highly heritable pattern (Vrieling et al. 1993). The F2 hybrid population consists of a set of genotypes that show a significant variation in PA diversity and concentration between plants. Thus, if PAs were to play a role in plant resistance, this should be reflected in differences throughout the set.

We compared the activity of specialist and generalist herbivores on the same genotypes of hybrid plants. For this, we designed two bioassays aimed at seeking answers to the following questions: 1) Is there any difference in the feeding damage to F2 hybrid plants produced by slugs and flea beetles? 2) If so, can its pattern be explained by the type and concentration of PAs? Additionally, considering that another three herbivores had been tested in an identical setup with the same set of genotypes, we analyzed the data from all the five herbivore bioassays and addressed the last question: 3) Does the feeding damage of the different herbivores covary with each other and is it related to the same or different PAs?

## Methods and Materials

### Study System

*Jacobaea vulgaris* is native to the Eurasian continent and invaded North America, Australia, and New Zealand (Doorduin et al. 2010). *Jacobaea aquatica*, a European endemic species, though closely related to *J. vulgaris* is not a sister species (Pelser et al. 2003). Based on morphology and molecular methods, putative hybrids between the two species have been found in Western and Central Europe (Chater and Walters 1976; Kirk et al. 2004). Thus, *Jacobaea* hybrids occur commonly in nature. The hybrids used in this study were developed in our lab (see Cheng et al. 2011a). In brief, two F1 offsprings were used to obtain 87 F2 individuals from a cross between *J. vulgaris* from a dune system, the Meijendel nature reserve (52°7′54″N, 4°19′46″E, The Netherlands) and *J. aquatica* from marsh area, the Zwanenwater nature reserve (52°48′38″N, 4°41′7″E, The Netherlands). The parents, F1 and F2 hybrids all are maintained in tissue culture and can be cloned ad libitum (see Plant Growth). The F2 hybrids were used for this study. *Jacobaea* plants are well known for their content of PAs, which have negative effects on the growth of fungal pathogens (Hol and van Veen 2002) and also play a dual role in plant-insect interactions (Cheng et al. 2011b, 2013; Macel 2003).

### Plant Growth

Plants were propagated by tissue culture and each of the 87 F2 individuals was cloned into six replicate individuals. Plants were potted in 0.8 L pots filled with a mixture of 95 % sandy soil (collected from Meijendel), 5 % potting soil (Slingerland Potgrond Company, Zoeterwoude, The Netherlands), and 1.5 g/L Osmocote slow release fertilizer (N:P:K = 15:9:11; Scott®, Scotts Miracle-Gro, Marysville, OH, USA). They were kept in a climate room (humidity 70 %, 16:8 hr/L:D, 20 °C/20 °C, light intensity 130 μm/m^2^/sec) for 6 weeks before starting the generalist herbivore bioassay. A total of 87 and 84 genotypes were used for the slug and flea beetle bioassay, respectively (in some cases fewer replicates survived). Some genotypes were represented by four or five replicates.

### Generalist Herbivore Bioassay

*Deroceras invadens* is a land slug that feeds on many different plant species. It is a significant pest in gardens, greenhouses, and pastures and is invasive in The Netherlands and many other parts of the world (Barker 2002). Slugs were collected in a lawn close to the Institute of Biology Leiden (52°09′27″N, 4°28′52″E, The Netherlands). The lawn was sprayed with water in the afternoon and covered with black plastic bags. The next morning *D. invadens* slugs hiding on the inner surface of the bags were collected and kept in a box with wet sand and fed with herbs and grasses from the same lawn. They were starved for 48 hr before the initiation of the bioassay. A subset of eight slugs was examined by Dr. Ton de Winter (malacologist at Naturalis Biodiversity Center, The Netherlands) who identified them all as *D. invadens*.

We used 2 cm diam leaf discs to conduct a no-choice bioassay. One slug and one leaf disc were placed on a layer of wet sand in a petri dish. Petri dishes were kept in the dark for 48 hr, after which the leaf disc was scanned to measure the percentage of eaten leaf disc. In the cases in which there were no signs of feeding, we replaced the *Jacobaea* leaf disc with a lettuce leaf disc and controlled it throughout 48 hr. It was observed that most slugs started eating the lettuce within the first 30 min. If the slug fed on the lettuce leaf disc, it was assumed that it refused to eat the *Jacobaea* leaf disc, and thus was recorded as zero. If the slug did not eat the lettuce leaf disc, it was assumed that other factors besides the food source could be affecting the non-feeding behavior of the slug, and it was considered as a missing value in the data analysis. This occurred four times in total. The experiment was conducted 6 times, each time using the same number of slugs as that of genotypes. In total, 498 slugs were used in the bioassay. Each slug and each plant were used only once.

### Specialist Herbivore Bioassay

*Longitarsus jacobaeae*, a flea beetle, is a specialist herbivore on *J. vulgaris*. This beetle is native to Eurasia and was introduced into US, New Zealand, and Australia as a ragwort biocontrol agent (Frick 1969; McLaren et al. 1999; Syrett et al. 1991). *Longitarsus jacobaeae* and the cinnabar moth *Tyria jacobaeae* have the same efficient *N*-oxidization enzyme to sequester PAs (Dobler et al. 2000; Narberhaus et al. 2003; Naumann et al. 2002), but they display different oviposition behavior and feed on *Jacobaea* plants in different metamorphic stages. *Longitarsus jacobaeae* lays eggs in the soil surrounding the host plants (Windig 1991), while *T. jacobaeae* lays eggs on the leaves. The adults of *L. jacobaeae* feed on leaves of *J. vulgaris*, leaving a characteristic “shot-hole” pattern (Frick 1969). Flea beetles were collected from *J. vulgaris* plants at the Meijendel nature reserve (52°7′548″N, 4°19′46″E, The Netherlands) with portable vacuum cleaners (DOMO DO211S, 14.4 V, Herentals, Belgium). After collection, they were kept in a climate room (humidity 70 %, 16:8 hr/L:D, 20 °C/20 °C, light intensity 130 μm/m^2^/sec) at the Institute of Biology Leiden, and fed with *J. vulgaris*. Each beetle was used only once for the bioassay.

We conducted a multiple-choice bioassay with whole leaves in a climate room (humidity 70 %, 16:8 hr/L:D, 20 °C/20 °C, light intensity 130 μm/m^2^/sec). Flea beetles were starved for 48 hr before initiating the experiment. First, a plastic box filled with floral foam was placed in an insect cage (Bug Dorm 32.5 × 32.5 × 77.0 cm). Water was added until the floral foam reached full water capacity. Then, the middle leaf of each genotype plant was cut with a scalpel and inserted into the floral foam in a random position in a grid, 5 cm apart. All the leaves kept fresh, and none wilted during the experiment. We released the same number of beetles as genotypes into the insect cage in each trial. The beetles were allowed to feed for 48 hr. The experiment was carried out six times, and 484 beetles were released in this bioassay. Herbivory was measured as the number of holes and leaf surface area consumed (mm^2^). Just before the experiment the fresh weight of the leaves was recorded as an indicator of plant size.

### Pyrrolizidine Alkaloid Data

Three to six replicates per genotype in F2 hybrids were analyzed by LC-MS/MS as described by Cheng et al. (2011a), and the mean PA values obtained were used for our analysis. In the F2 hybrid cross, 37 individual PAs were detected (Cheng et al. 2011a). Based on their presumed biosynthesis, PAs have been classified into four main groups: senecionine-, jacobine-, erucifoline-, and otosenine-like PAs (Cheng et al. 2011a; Pelser et al. 2003). These alkaloids exist in two forms: tertiary amine and *N*-oxide (Hartmann 1999). A large variation in the concentration of *N*-oxides (267.4 to 3927.9 μg/g) has been reported in F2 hybrid shoots (Cheng et al. 2011a).

The PA concentration and composition have proved to possess a high narrow sense heritability (Vrieling et al. 1993). To further confirm that PA concentration is strongly genotype-dependent, we chose 20 genotypes from the F2 hybrids that had also been used by Cheng et al. (2011a) and grew them under the same conditions (Cheng et al. 2011a). The PA content was determined again by LC-MS/MS. The results showed that the PAs of the plant measured by Cheng et al. (2011a) were highly correlated with the values obtained for this independent set of genotypes. There was a high correlation between the total PA concentration (Spearman correlation: *r* = 0.7, *P* = 0.001) and that of four groups of PAs (Spearman correlation: *r* > 0.7, *P* < 0.001) of the two sets of plants with the same genotypes.

### Statistical Analysis for Slug Bioassay

We used a general linear model to check whether the trials influenced the feeding damage of the slug *D. invadens*. The percentage of feeding damage was used as the dependent variable, and trials were considered as a random factor. The percentage of feeding damage was arcsine square root transformed to achieve normality. Normality was confirmed by Shapiro-Wilk tests. As there were significant differences among trials, we decided to use the unstandardized residuals (slug feeding residual, SFR) for further tests. General linear models then were performed on SFR with plant genotype as a random factor to determine whether slug resistance differed among the F2 hybrids.

We calculated genotypic mean SFR and used these in linear multiple regression to test whether the four structural groups of PAs were correlated with slug feeding. The genotypic mean concentrations of the four structural PA groups were log transformed and considered as independent variables.

Only the concentration of spartioidine and spartioidine N-oxide were normal distributed, so all the other individual PAs were log transformed to achieve normality. However, even after log transformation, five PAs were not normally distributed, and in these cases we used a Spearman rank correlation test. In all other cases, a two-tailed Pearson correlation test was applied. To correct for multiple testing, the *P*-values of the individual tests were adjusted with the sequential Bonferroni method.

### Statistical Analysis for Flea Beetle Bioassay

Two variables were used to measure flea beetle feeding damage: the number of holes and the total area consumed (mm^2^). As both variables were highly correlated with each other (*R* ^*2*^ = 0.884, *N* = 484, *P* < 0.001), only the total area consumed was used for further analysis.

We applied a general linear model to correct the data for differences among trials and used the unstandardized residuals (beetle feeding residual, BFR). In this model, feeding damage by the flea beetle was defined as the dependent variable, and the trial was defined as the random factor. We also used general linear models to determine whether feeding damage by the flea beetle differed in relation to the plant genotype. The BFR was considered as the dependent variable, and plant genotype as the random factor with the fresh weight of the leaf as a covariate.

A multiple regression analysis was conducted to determine whether the four structural groups of PAs affected the feeding choice of flea beetles. The BFR was considered as the dependent variable. The genotypic mean concentrations of the four structural PAs groups and the leaf fresh weight were used as independent variables. The PA concentrations were log transformed. Two-tailed Pearson or Spearman rank correlation tests were conducted between BFR and the concentrations of individual PAs.

### Comparison of Proxies for Herbivore Feeding

Three other herbivores have been tested using the same set of F2 genotypes: the specialist lepidopteran cinnabar moth *Tyria jacobaeae* (oviposition bioassay), the generalist thrips *Frankliniella occidentalis* (silver damage bioassay), and the generalist leaf miner *Liriomyza trifolii* (pupae number bioassay) in previous studies (Cheng et al. 2011b, 2013; Cheng 2012). Two-tailed Pearson correlation tests were performed to check for correlations among the different bioassays. The BFR corrected for leaf fresh weight and SFR were used for the feeding damage of flea beetles and slugs, respectively. In the case of the cinnabar moth oviposition bioassay, egg batch numbers were corrected for cage number. The silver damage was log transformed for normality, and the number of leaf miner pupae was corrected for plant size. The Pearson correlations between herbivore feeding estimates and PAs were summarized. The concentration of total tertiary amines, total *N*-oxides, and four groups of PAs were also log transformed.

All analyses were conducted in SPSS 19.0.

## Results

### Slug Herbivore Feeding Damage and Response to Pyrrolizidine Alkaloids

Slug feeding damage was below 60 % in all F2 genotypes and below 30 % in 67 of them (Fig.S1a). As feeding of the slugs differed among trials (*F*_*5*, *492*_ = 5.68; *P* < 0.001) we used slug feeding residuals (SFR) corrected for trials for the next tests. The SFR were genotype-dependent (*F*_*86*, *411*_ = 2.69; *P* < 0.001).

**Fig. 1:**
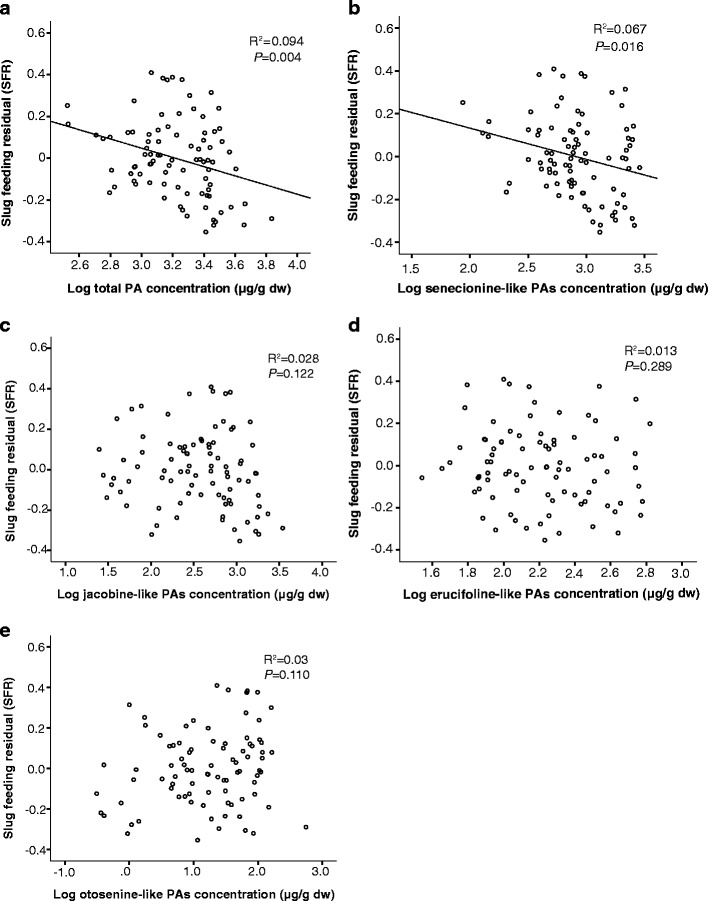
Scatter plots of the slug arcsine square root-transformed percentage of feeding damage corrected for trial (SFR) and the concentration of total pyrrolizidine alkaloids (PAs) (**a**), senecionine-like PAs (**b**), jacobine-like PAs (**c**), erucifoline-like PAs (**d**), otosenine-like PAs (**e**) of 87 F2 hybrids of *Jacobaea vulgaris* and *Jacobaea aquatica*. All PAs were log transformed to obtain normal distribution. Each dot represents the genotypic mean value of one of the 87 F2 genotypes

A significant negative correlation was observed between the total PA, *N*-oxides, and senecionine-like PAs concentrations and SFR (Fig. 1a and b), while the other groups of PAs had no significant correlation with SFR (Fig. 1c, d and e). The multiple regression analysis also showed that senecionine-like PAs had a significant negative effect on SFR. In contrast to the simple correlation among the four structural PA groups and SFR, the otosenine-like PAs had a positive effect on SFR, while the other two groups exerted no effect (Table 1).

**Table 1:** Multiple regression of the slug feeding residual (SFR) against the sum concentration of the four main structural groups of pyrrolizidine alkaloids (PAs, µg/g dw) in the host plants of 87 F2 hybrid genotypes from a cross between *Jacobaea vulgaris* and *Jacobaea aquatica*. For the regression model: adjusted *R* ^*2*^ = 0.0973; *F*_*4*, *82*_ = 3.318; *P* = 0.014

Predictors^a^	Estimate	*t* value
(Intercept)	−0.001	−0.062
Sn	−0.049	−2.479*
Jb	−0.030	−1.556
Er	−0.007	−0.354
Ot	0.042	2.195*

*P < 0.05

^a^Sn, Jb, Er, and Ot are log transformed concentration of senecionine-, jacobine-, erucifoline-, and otosenine- like PAs

After correction for the number of tests by the sequential Bonferroni test, two-tailed Pearson or Spearman rank correlation tests showed that SFR was negatively correlated with the concentration of seneciphylline, seneciphylline *N*-oxide, spartioidine, spartioidine *N*-oxide, senecivernine, and jacozine *N*-oxide (Table S1, Fig.S1).

### Flea Beetle Preference and Response to PAs

The genotypic mean leaf area removed by flea beetle feeding ranged from 0 to 25 mm^2^ (Fig.S1b). As the feeding damage of the flea beetle differed among trials (*F*_*5*, *478*_ = 4.147; *P* = 0.001), we used flea beetle feeding residual (BFR) corrected for trials for the next tests.

While BFR did not differ among plant genotypes (*F*_*83*, 397_ = 1.098; *P* = 0.277), leaf fresh weight had a positive effect on the BFR (*F*_*1*, *397*_ = 9.084; *P* = 0.003).

**Fig. 2:**
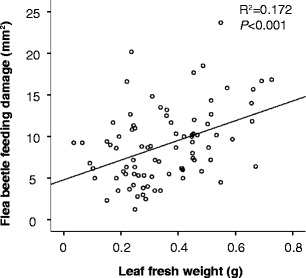
Scatter plot of the genotypic mean of leaf fresh weight and genotypic mean of flea beetle feeding damage. Each dot is the mean of 4–6 replicates of 84 F2 genotypes

Multiple regression analysis showed a significant positive correlation between BFR and leaf fresh weight (Table 2, Fig. 2), but there were no significant correlations among the four structural groups of PAs and BFR (Table 2).

**Table 2:** Multiple regression of flea beetle feeding residual (BFR) corrected for trials against the sum concentration of four structural groups of pyrrolizidine alkaloids (PAs, µg/g dw) in the 84 F2 hybrid genotypes from a cross between *Jacobaea vulgaris* and *Jacobaea aquatica*. For the regression model: adjusted *R* ^*2*^ = 0.2425; *F*_*15*, *68*_ = 2.771; *P* = 0.002

Predictors^a^	Estimate	*t* value
(Intercept)	−5.773	−1.112
Fw	11.680	3.706**
Sn	0.234	0.162
Jb	−0.117	−0.126
Er	0.276	0.180
Ot	0.492	0.771

**P < 0.01

^a^Sn, Jb, Er, Ot are log transformed concentration of senecionine-, jacobine-, erucifoline-, and otosenine- like PAs. Fw is the genotypic mean of leaf fresh weight

No correlation was observed between the individual PAs and BFR (data not shown). There was, however, a significant positive correlation with the fresh weight of the leaf (*R* ^*2*^ = 0.172, *P* < 0.001) (Fig. 2).

### Comparisons of Proxies for Herbivore Feeding and Pyrrolizidine Alkaloids

Silver damage by thrips was positively correlated with slug feeding damage (Table 3). No other significant correlations between proxies for herbivore feeding were found among the five tested herbivores.

**Table 3:** Pearson correlation tests among five different herbivore feeding^a^ bioassays of *Jacobaea* F2 hybrids from a cross between *Jacobaea vulgaris* and *Jacobaea aquatica*

	Specialist herbivores		Generalist herbivores		
	Flea beetle (*N* = 87)	Cinnabar moth (*N* = 35)	Slug (*N* = 84)	Thrips (*N* = 98)	Leafminer (*N* = 90)
Flea beetle					
Cinnabar moth	0.057				
Slug	−0.191	−0.136			
Thrips	−0.101	−0.047	0.334**		
Leafminer	0.094	−0.189	−0.091	−0.095	

**P < 0.01

^a^Proxies for herbivore feeding used: flea beetle feeding residual, corrected for leaf fresh weight, for the flea beetle (*Longitarsus jacobaeae*) bioassay; the number of egg batches, corrected for cage number, for the cinnabar moth (*Tyria jacobaeae*) oviposition bioassay; the slug feeding residual (SFR) for the slug (*Deroceras invadens*) bioassay; the log transformed silver damage for the Western flower thrips (*Frankliniella occidentalis*) bioassay and the number of pupae, corrected for plant size, for the American serpentine leafminer (*Liriomyza trifolii*) bioassay

There were positive or negative correlations between herbivore feeding and the concentration of PAs in all bioassays with the exception of flea beetles (Table 4). The specialist herbivores showed either a positive correlation (cinnabar moth) or no correlation at all with the PA concentration (flea beetle). The concentration of at least one type of PAs was negatively correlated with the proxies of generalist herbivores. However, the feeding behavior of generalists was related to different structural groups of PAs. The slug preferred to feed on leaves with small amounts of senecionine-like PAs. Thrips silver damage was lower in the leaves with high jacobine-like Pas, and more pupae of the leaf miner were found on the leaves with low otosenine-like PAs.

**Table 4:** Summary of Pearson correlations between pyrrolizidine alkaloid (PA) concentrations and the proxies of herbivore feeding^a^ in the five bioassays conducted with *Jacobaea* F2 hybrids from a cross between *Jacobaea vulgaris* and *Jacobaea aquatica*

PA	Specialist herbivores		Generalist herbivores		
	Flea beetle (*N* = 87)	Cinnabar moth (*N* = 35)	Slug (*N* = 84)	Thrips (*N* = 94)	Leafminer (*N* = 90)
Total tertiary amines	0.079	0.391*	−0.206	−0.266**	−0.235*
Total N-oxide	0.042	0.043	−0.283**	−0.282**	−0.060
Senecionine-like PAs	0.030	−0.042	−0.259*	−0.204*	−0.091
Jacobine-like PAs	0.017	0.350*	−0.167	−0.297*	−0.188
Erucifoline-like PAs	−0.004	−0.078	−0.115	−0.159	0.226*
Otosenine-like PAs	0.088	0.303	0.173	0.030	−0.267*

*P < 0.05, **P < 0.01

^a^The feeding damage data used is the same as that in Table 3

## Discussion

The results of this study revealed that PAs, one of the major groups of SMs of *Jacobaea* hybrid plants, deterred the generalist herbivore *D. invadens*. We also observed that the feeding damage inflicted by this generalist slug was genotype-dependent, a fact that could be explained partly by the genotype-dependent PA concentrations. Cheng et al. (2011a) had shown that PA concentrations varied widely with the genotype. A negative correlation was observed for senecionine-like PAs, and considering that half of total *N*-oxide concentration is derived from senecionine-like PAs, a correlation was established between slug feeding damage and total *N*-oxide concentration.

Among the 17 senecionine-like PAs, seneciphylline, seneciphylline *N*-oxide, spartioidine, spartioidine *N*-oxide, and senecivernine were negatively correlated with slug feeding (Table S1). It is important to note that there is a significant difference among the concentration of these five PAs. The concentration of seneciphylline *N*-oxide is more than ten times that of the other four PAs combined. Interestingly, all these PAs have a double bond between C13 and C19 except for senecivernine (Fig.S3). In fact, eight of the senecionine-like PAs have a double bond at C13, four of which were found to be negatively correlated with slug feeding. These PAs are highly correlated with each other and also correlated with the other senecionine-like PAs that do not have the C13 double bond. Apart from this type of PAs, three jacobine-like PAs also have a double bond at C13 and are negatively correlated with slug feeding. These three PAs were significantly correlated with those senecionine-like PAs with a double bond at C13, while they were not correlated to senecionine-like PAs without this double bond. These observations strongly suggest that the presence of this double bond is related to the slug deterrence.

The multiple regression analysis of *D. invadens* showed that otosenine-like PAs had positive effects on slug feeding. Even though we did not find significant simple correlations between slug feeding and the total otosenine-like (*r* = 0.173) or individual otosenine-like PAs (Table S1), the correlation coefficient was significantly positive. Furthermore, the concentration of otosenine-like PAs is relatively low, often below 2 % of the total PA content.

In this study, the specialist *L. jacobaeae* preferred feeding on larger leaves. Adult herbivory by flea beetles has been reported to be proportional to plant size in the field (Windig 1993). Specialist herbivory often is restricted to one type of food source, and they may easily face food shortage, so it is a useful strategy to feed on big plants. Plant size also was shown previously to be a significant factor in the oviposition choice for the specialist moth *T. jacobaeae* (Cheng et al. 2013).

A previous study had revealed that there was no significant positive correlation between the cinnabar moth oviposition preference of *J. vulgaris* plants and their PA content (Macel et al. 2002). However, that study included only a limited number of PAs. With the help of more sensitive technology and extended analyses, including measurements of the tertiary amine and *N*-oxide forms, Cheng et al. (2013) was able to show that *T. jacobaeae* preferred laying eggs on plants with higher amounts of tertiary amines of jacobine-like PAs. *Longitarsus jacobaeae* has not been as extensively studied as *T. jacobaeae*. Kirk et al. (2012) studied the feeding preference of several *Longitarsus* species, including *L. jacobaeae*, on a range of different *Jacobaea* and *Senecio* plants. Their conclusion that the PA content in plants did not affect flea beetle feeding preference is consistent with our observations on the absence of correlations with the genotype and with the four structural PA groups. However, even though *L. jacobaeae* is apparently not attracted by PAs, it does sequester them (Dobler et al. 2000).

As mentioned above, Cheng et al. (2013) conducted an oviposition bioassay using the specialist *T. jacobaeae* and found that they preferred plants with higher amounts of tertiary amines of jacobine-like PAs. In the bioassay with the generalist *Frankliniella occidentalis*, feeding damage has been reported to be negatively correlated with the total PA content and with that of jacobine-like PAs (Cheng et al. 2011b). The *Liriomyza trifolii* pupae bioassay has revealed a weak negative correlation between the number of pupae and the concentration of otosenine-like PAs (Cheng 2012). Our study showed that senecionine-like PAs deterred the feeding of the slug *D. invadens*. Therefore, it appears that different groups of PAs are attractive or deterring to different herbivores. Collectively, these findings suggest that PA diversity is maintained by the selection of different generalist and specialist herbivores.

Comparing the different bioassays, we found a significant positive correlation only between slug feeding damage and thrips silver damage (Table 3). There were no other significant correlations. It is noteworthy that five over six correlations between the feeding damage of generalist and specialist herbivores were negative (Table 3). The results showed with the exception of the flea beetle, the amount of herbivore damage was genotype-dependent (Table 4), suggesting that there are multiple resistance mechanisms against herbivores in *Jacobaea* plants. In general, only about 10 % of the variance in resistance can be explained by PAs implying that other secondary or primary metabolites, or physical barriers also may play a role (Nuringtyas et al. 2012).

Due to practical issues, we designed no-choice and multiple-choice bioassays. Many studies have revealed that both approaches often yield the same results. For instance, *L. jacobaeae* feeding preferences in a multiple-choice experiment were highly correlated with the results from a no-choice experiment (Spearman rank correlation: *r* = 0.779, *N* = 17, *P* < 0.001) (Kirk et al. 2012). In addition, the feeding damage of thrips (*Frankliniella occidentalis*) on F2 hybrids (on a subset of the F2 hybrids plants of our study) from a choice bioassay was consistent with the results obtained in a non-choice bioassay (Leiss et al. 2009). It is, thus, most likely that the approach did not affect our conclusions.

The *Jacobaea* F2 hybrids study system already has been used in a number of herbivore bioassays (Cheng et al. 2011b, 2013; Cheng 2012; Leiss et al. 2009). Hybridization has been proved to lead to a greater quantitative and qualitative variation of SMs (Cheng et al. 2011a; Fritz 1999; Orians 2000). The other advantage of using an F2 progeny is that specific traits can be studied against an average equal genetic background (Hochwender et al. 2000; Lexer et al. 2003). The considerable and novel PA diversity present in *Jacobaea* F2 hybrids can lead to variations in the performance of insect herbivores (Cheng et al. 2011a; Macel 2003; van Dam et al. 1995). Hybrids are, thus, useful to investigate the functions of plant secondary metabolites in resistance to herbivores (Cheng et al. 2011c).

In summary, our results showed that the generalist herbivore slug was deterred by senecionine-like PAs, but the PA content in *Jacobaea* plants did not affect the specialist herbivore flea beetle. Leaf fresh weight was positively correlated with flea beetle feeding, suggesting that plant size could be more important than PA concentration for specialist herbivores. Altogether, the results of the current experiment combined with previous herbivore tests on the same experimental system (Cheng et al. 2011b, 2012, 2013) produced only partial evidence to support the Generalist-Specialist dilemma. The concentration of at least one PA type was negatively correlated with generalists, while the specialist caterpillar showed positive correlations with some PA types but no significant correlations with the specialist flea beetle. Additionally, we found no negative correlations between specialist and generalist feeding. In part, this may be caused by unknown factors that influence specialists and generalists in the same way. Thus, although the Generalist-Specialist dilemma was only partly proved here, our results clearly revealed that the divergent selection pressure from the different insect herbivores are important for the evolution and function of PA diversity.

## Electronic supplementary material

Below is the link to the electronic supplementary material.

ESM 1 (DOCX 2249 kb)
