# Supplementary material for: Testing the Generalist-Specialist Dilemma: The Role of Pyrrolizidine Alkaloids in Resistance to Invertebrate Herbivores in Jacobaea Species
Source: J Chem Ecol. 2015 Feb 11;41(2):159–67. doi: 10.1007/s10886-015-0551-4 (PMC4351440; doi:10.1007/s10886-015-0551-4)
Supplement: Supplementary file 1 — (DOCX 2249 kb) [file 10886_2015_551_MOESM1_ESM.docx]

**Supplementary Material**

**Table S1** Two tailed Pearson/Spearman correlation tests between slug feeding residual (SFR) and the concentrations of individual pyrrolizidine alkaloids (PAs, µg/g dw) in the 87 F2 genotypes from a cross between *Jacobaea vulgaris* and *Jacobaea aquatica*

| Group | PA | r/r_s_ | *P* |
| --- | --- | --- | --- |
|  | senecionine | -0.201 | 0.062 |
|  | senecionine *N*-oxide | -0.184 | 0.087 |
|  | integerrimine | -0.160 | 0.139 |
|  | integerrimine *N*-oxide | -0.131 | 0.227 |
|  | retrorsine | -0.125 | 0.249 |
|  | retrorsine *N*-oxide | -0.005 | 0.962 |
|  | usaramine | -0.112 | 0.300 |
| Senecionine-like PAs | usaramine *N*-oxide^a^ | -0.041 | 0.706 |
|  | riddelliine | 0.205 | 0.057 |
|  | riddelliine *N*-oxide | -0.124 | 0.251 |
|  | seneciphylline | -0.374 | <0.001* |
|  | seneciphylline *N*-oxide | -0.342 | 0.001* |
|  | spartioidine | -0.380 | <0.001** |
|  | spartioidine *N*-oxide | -0.345 | 0.001* |
|  | acetylseneciphylline | 0.068 | 0.531 |
|  | acetylseneciphylline *N*-oxide | 0.638 | 0.265 |
|  | senecivernine | -0.341 | 0.001* |
|  | jacobine | -0.113 | 0.297 |
|  | jacobine *N*-oxide^a^ | -0.171 | 0.114 |
|  | jacoline | -0.100 | 0.356 |
|  | jacoline *N*-oxide^a^ | -0.156 | 0.149 |
| Jacobine-like PAs | jaconine | -0.093 | 0.394 |
|  | jaconine *N*-oxide^a^ | -0.159 | 0.142 |
|  | jacozine | -0.238 | 0.027 |
|  | jacozine *N*-oxide | -0.336 | 0.001* |
|  | dehydrojaconine | -0.259 | 0.015 |
|  | erucifoline | -0.095 | 0.384 |
| Erucifoline-like PAs | erucifoline *N*-oxide | -0.137 | 0.204 |
|  | acetylerucifoline | 0.045 | 0.682 |
|  | acetylerucifoline *N*-oxide | 0.011 | 0.920 |
|  | senkirkine^a^ | -0.002 | 0.985 |
|  | otosenine | 0.163 | 0.131 |
| Otosenine-like PAs | onetine | 0.123 | 0.256 |
|  | desacetyldoronine | 0.137 | 0.207 |
|  | florosenine | 0.141 | 0.195 |
|  | floridanine | 0.088 | 0.426 |
|  | doronine | 0.161 | 0.142 |

^a^ Spearman correlation tests were carried out for PAs with not normally distributed concentrations

**P*<0.05, ***P*<0.01indicates significance after the sequential Bonferroni test


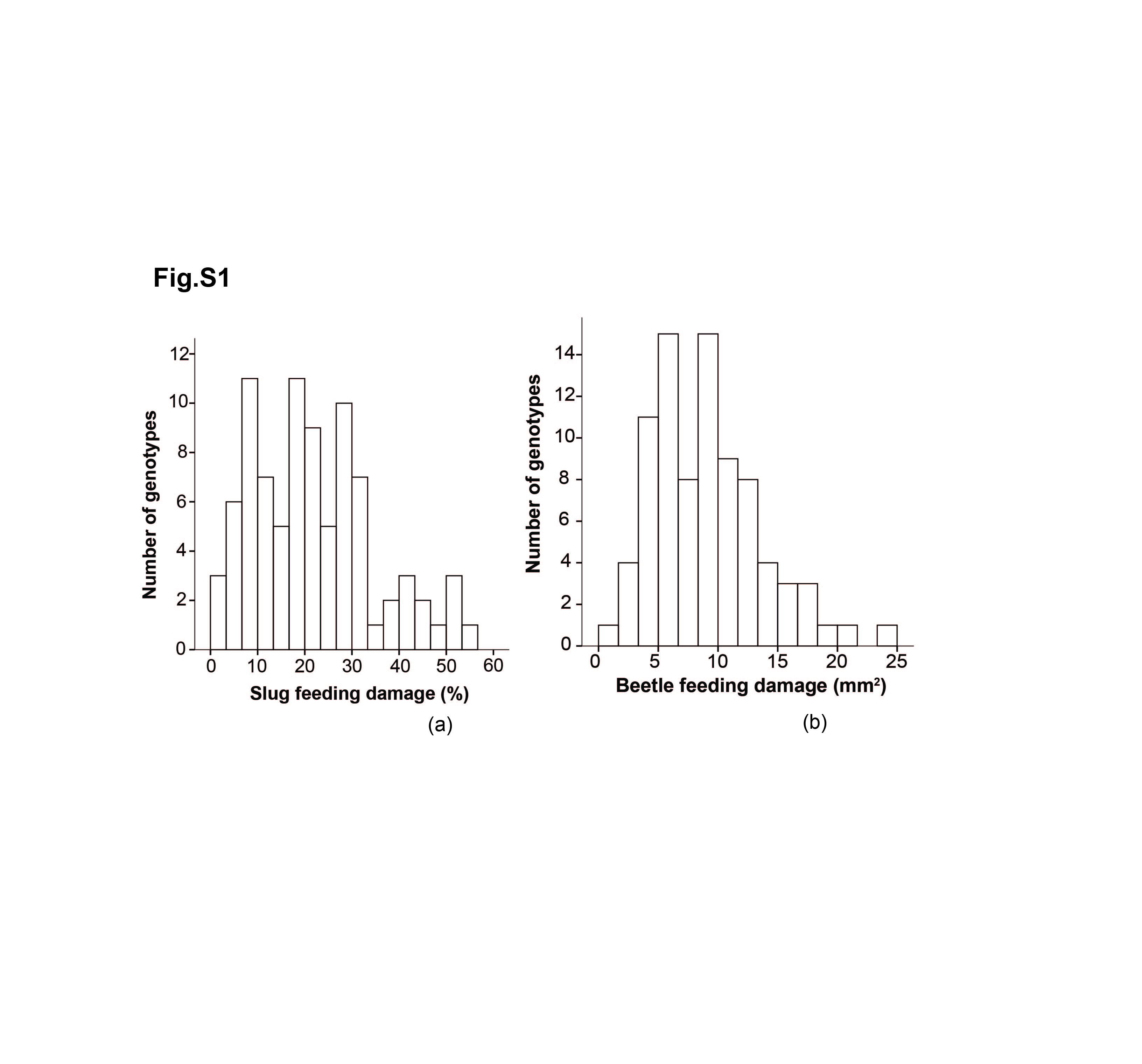


**Fig.S1** (**a**) Frequency distribution of genotypic mean percentage of slug feeding damage of 87 F2 hybrids. Each genotype is the mean of 4-6 replicates. In total, 498 plants were used in the slug bioassay. (**b**) Frequency distribution of the genotypic mean square millimeter of flea beetle feeding damage of 84 F2 hybrids. Each genotype is the mean of 4-6 replicates. In total, 484 plants were used in the flea beetle bioassay


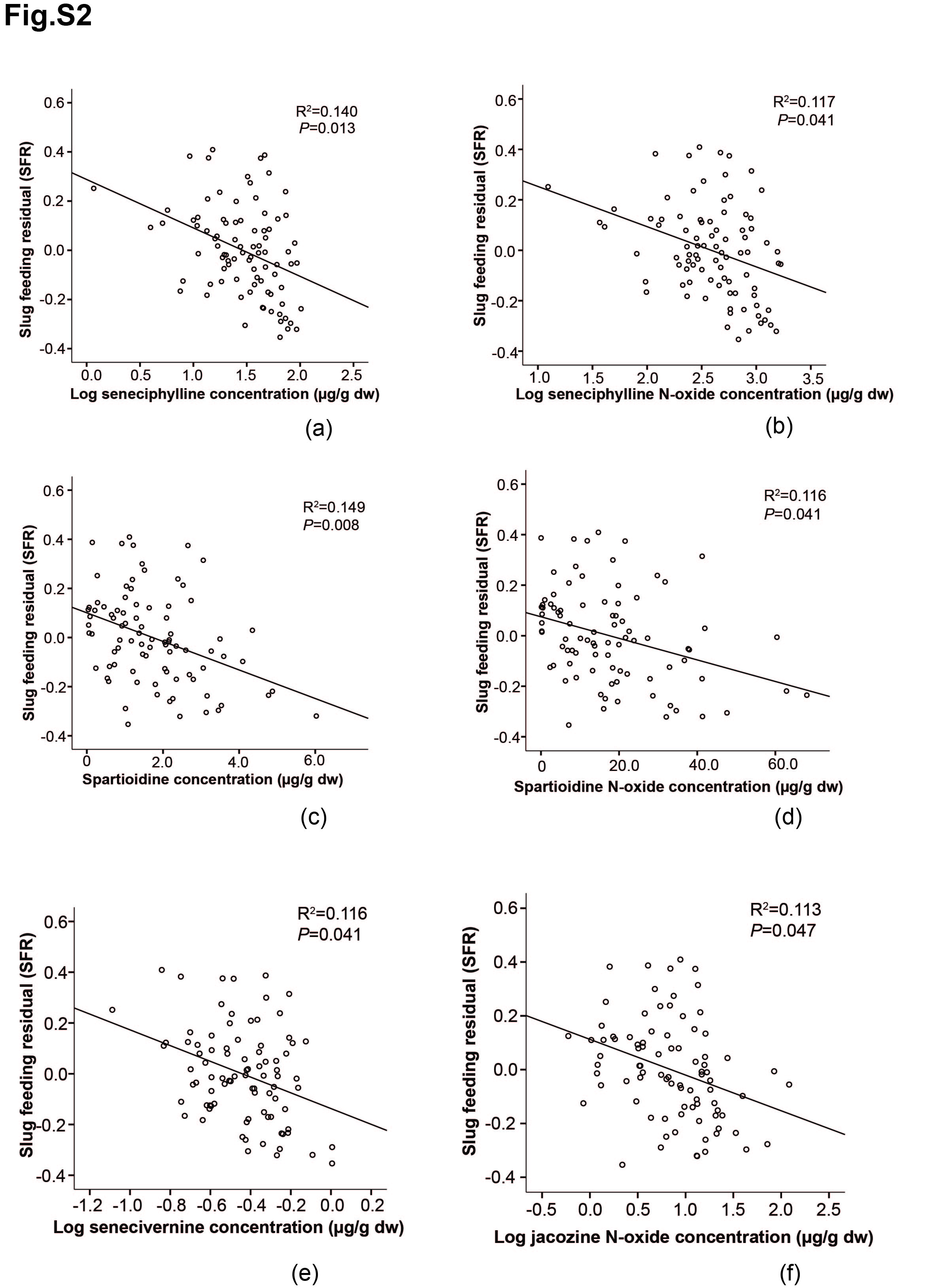


**Fig.S2** Scatter plots between slug arcsine square root transformed percentage of feeding damage corrected for trial (SFR) and the concentration of individual pyrrolizidine alkaloids (PAs): seneciphylline (**a**), seneciphylline N-oxide (**b**), spartioidine (**c**), spartioidine N-oxide, (**d**), senecivernine (**e**), jacozine N-oxide (**f**) of 87 F2 hybrids of *Jacobaea vulgaris* and *Jacobaea aquatica*. The genotypic mean of PA concentrations (except for spartioidine and spartioidine N-oxide) were log transformed. Each *P* value was corrected by sequential Bonferroni test. Each dot represents the genotypic mean value of one of 87 F2 genotypes


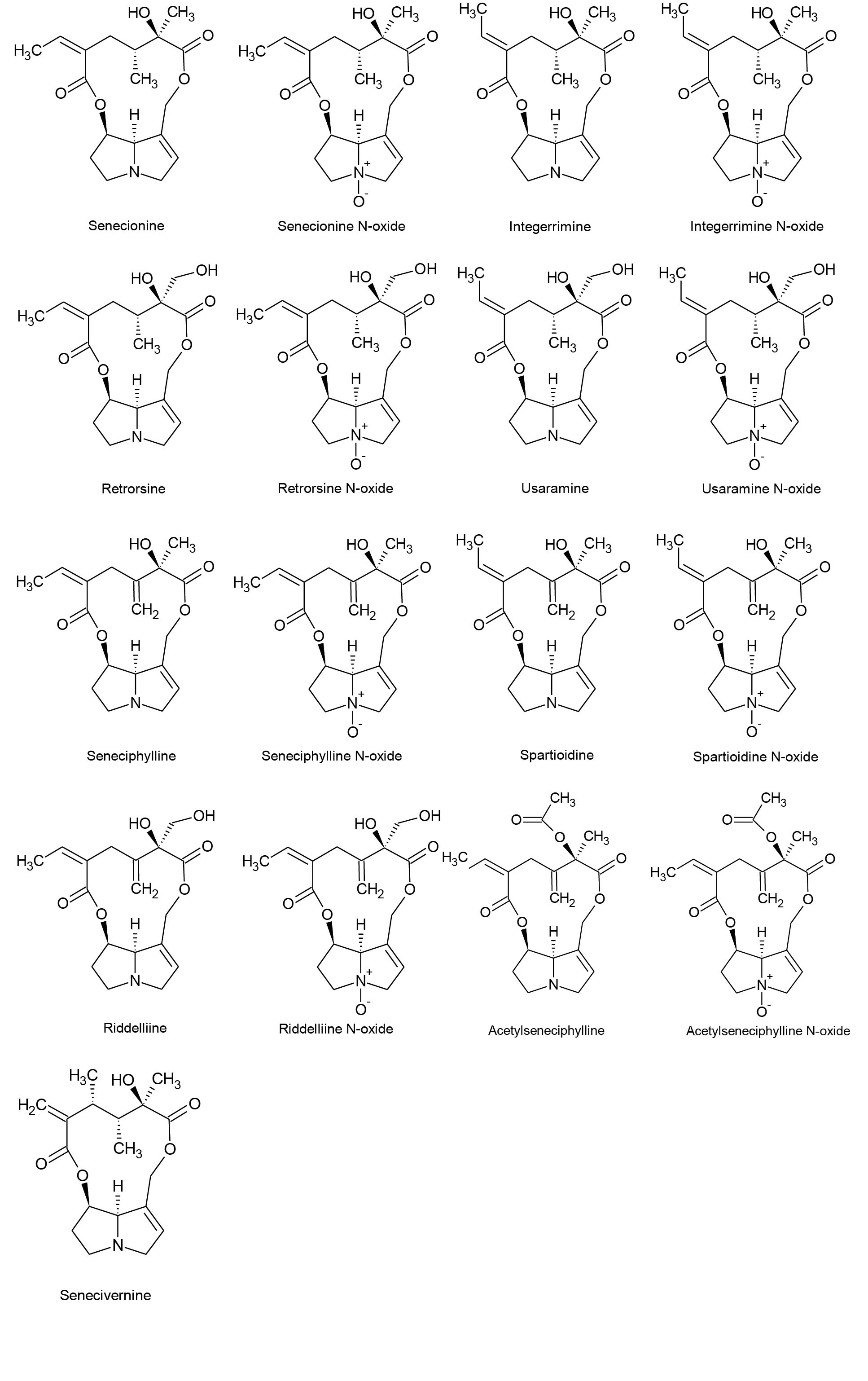


**Fig.S3** Chemical structures of senecionine-like PAs
